# Effect of Carbon Addition and Mixture Method on the Microstructure and Mechanical Properties of Silicon Carbide

**DOI:** 10.3390/ma13173768

**Published:** 2020-08-26

**Authors:** Zeynep Aygüzer Yaşar, Richard A. Haber

**Affiliations:** 1 Department of Metallurgical and Materials Engineering, Hitit University, Corum 19030, Turkey; zayguzeryasar@gmail.com; 2 Department of Material Science and Engineering, Rutgers, The State University of New Jersey, Piscataway, NJ 08854, USA

**Keywords:** silicon carbide, carbon, mixture method, microstructure, spark plasma sintering, ceramics mechanical property

## Abstract

High dense (>99% density) SiC ceramics were produced with addition of C and B_4_C by spark plasma sintering method at 1950 °C under 50 MPa applied pressure for 5 min. To remove the oxygen from the SiC, it was essential to add C. Two different mixture method were used, dry mixing (specktromill) and wet mixing (ball milling). The effect of different levels of carbon additive and mixture method on density, microstructure, elastic modulus, polytype of SiC, Vickers hardness, and fracture toughness were examined. Precisely, 1.5 wt.% C addition was sufficient to remove oxide layer from SiC and improve the properties of dense SiC ceramics. The highest hardness and elastic modulus values were 27.96 and 450 GPa, respectively. Results showed that the 4H polytype caused large elongated grains, while the 6H polytype caused small coaxial grains. It has been observed that it was important to remove oxygen to achieve high density and improve properties of SiC. Other key factor was to include sufficient amount of carbon to remove oxide layer. The results showed that excess carbon prevented to achieve high density with high elastic modulus and hardness.

## 1. Introduction

Silicon carbide (SiC) is widely used in the defense industry (bulletproof vests), turbine engines, nozzles, heat conducting tube, and aerospace applications [1,2,3,4,5,6,7]. Since SiC has remarkable properties such as low theoretical density (3.21 g/cm^3^), a high hardness, a high elastic modulus, high thermal conductivity, good wear and oxidation resistance, and low coefficient of thermal expansion [2,3,8,9,10,11,12,13,14,15,16,17]. The problem with SiC is that it is really difficult to achieve high dense bodies due to its strong covalent bonding and low self-diffusion coefficient without using sintering aids for instance B_4_C, B, Al, Si, TiC, ZrB_2_, TiB_2_, Al_2_O_3_, Y_2_O_3_, and C or pressure-assisted sintering method [3,6,9,13,18,19,20,21,22,23,24,25,26,27,28]. Depending on particle size of powder, moisture in the air and additives, nonoxide high-temperature materials like B_4_C, SiC, and TiB_2_ tend to have oxide layer on their surfaces. This oxide layer inhibits to achieve high density and causes grain coarsening [8,9,29,30,31]. Adding C to SiC eliminates the oxygen problem, and helps to obtain high dense ceramics [22,23,25,26,32]. SiC ceramics can be densify by pressureless sintering, hot pressing (HP), and spark plasma sintering (SPS) with solid state or liquid phase sintering [3,11,20,21,27,33]. SPS is a quite new technique that allows sintering of materials without exaggerated growth in a short time [3,6,7,15,34,35,36,37].

The aim of this study is to optimize the properties of SiC by adding different amounts of carbon to SiC and mixing powders with different methods. To achieve these goals, from 0.5 to 5.0 wt.% C and 0.5 wt.% B_4_C were added to SiC, and powders were mixed with dry mixing and wet mixing. Dense SiC ceramics were produced by spark plasma sintering method.

## 2. Materials and Methods

In this research, the starting powder was commercially available submicron α-silicon carbide (SiC) (UF-25, H. C. Starck GmbH & Co., Munich, Germany) with the major impurity being oxygen 1.7 wt.%, and trace amounts of the Fe, Al, and Ca. The chemical and physical characteristics of SiC powder can be seen in Table 1. It was provided by the manufacturer. HC. Starck boron carbide (HD-20, H. C. Starck GmbH & Co., Germany) and carbon (Lamp black from Fisher Scientific, Walsham, MA, USA) were used as sintering aids.

The effect of the mixture method on the microstructural and mechanical properties of the silicon carbide was investigated using two different methods which were dry mixing by Specktromill (Spex Inc., Los Angeles, CA, USA, 8000M) and wet mixing by ball milling.

For the dry mixing series, 6.5 g of mixture were prepared with 0.5 wt.% B_4_C, 0.5–5 wt.% with 0.5 increment C and SiC. Powders were weighted and place in a small bottle without media and blended with Specktromill for 5 min.

For the wet mixing series, 20 g of dry powders mixtures were prepared with 0.5 wt.% B_4_C and 0.5–5 wt.% with 0.5 increment C and SiC. Powders were weighted and put into a Nalgane bottle with SiC ball and ball milled for 24 h. After that, to separate the media from the liquid mixture, the slurries were sieved using 1.4 mm mesh sieve. The liquid mixture had been put on a hot plate at 275 °C and allowed to dry. Then, the powder chunks were ground with mortar and pestle to prevent agglomeration.

For densification, 6.5 g of every powder mixture were used for one sample. The powder was placed in a graphite die with an internal diameter of 20 mm. Graphite foil was lined between powders and the die as well as the punches to avoid samples reacting with the die set. Then, samples were densified with two-stage sintering using spark plasma sintering (SPS, Thermal Technology LLC. Model 10-4, Santa Rosa, CA, USA). The SPS was heated to 1400 °C with a 200 °C/min heating rate under vacuum with a 50 MPa applied pressure. The SPS was held for 1 min intermediate dwell to allow the carbon to react with oxide layer on the SiC, and then, the SPS was heated to 1950 °C with 200 °C/min heating rate under 50 MPa applied pressure and held for 5 min. After dwelling for 5 min at sintering temperature, SPS furnace was naturally cooled down to room temperature.

After sintering, the dense samples required sandblasting to remove excess graphite foil from the surface. Then, to produce a smooth and clean surface, both sides of dense samples surface were ground down using a surface grinder with a 120-grit diamond wheel with a lubricant:water solution (1:20 vol%) to keep the specimen surface wet while grinding to prevent possible damage from friction.

After that, the density of sintered samples was measured with Archimedes principle using DI water as the immersion medium and elastic properties were measured using ultrasound analysis method. Polytype of SiC was determined using X-ray diffraction. Phase identification and quantification were estimated via a PANalytical X’Pert X-ray diffraction unit, and a Cu Kα X-ray source at 45 kV and 40 mA was used for analysis. Scan range was from 10° to 90° 2θ. The virtual step size was 0.0131° and rotation was 16 rpm for each scan. All scans were recorded by Data Collector, and after that, Rietveld analysis was done using MDI Jade 9 software to determine the phase of the SiC. Especially, the free carbon peak present at 26.6° 2θ was important to understand if the addition of carbon was reacted during sintering processing or not.

Using LECO Vari/Cut 50 diamond saw, the samples were sectioned and mounted in epoxy using a Buehler SimpliMet 1000 Mounting Press. Then, samples were polished to 0.25 µm finish using Buehler EcoMet 250 Grinder-Polisher. To highlight SiC grain boundaries, one piece of polished samples was etched by a modified Murakami method (20 g KOH and 20 g K_3_Fe(CN)_6_ in 60 mL DI water).

Field emission scanning electron microscope (Zeiss Sigma FESEM with Oxford EDS System) was used to examine pores, inhomogeneity, and shape of grains. Average grain sizes of the dense samples were estimated by linear intercept procedure using minimum of 100 intersections. Microhardness was performed on polished surfaces using LECO M-400-G3 microhardness tester with Vickers diamond tip. Specifically, 9.8 N load was applied for 10 s on polished sample surfaces. At least 10 acceptable indentations were made. Via Keyence VHX 5000 digital microscope, indent sizes were measured.

According to the following equation, Vickers hardness value was calculated to refer to ASTM C1327-15 [38]:

(1) $$H_{v}\left(\mathrm{GPa}\right)=1.8544\frac{P}{d^{2}}$$

where *P* (kgf) is a force and *d* (mm) is the average length of the two diagonals of the indentation. Each hardness value presents the mean of the 10 calculated hardness.

The indentation fracture can be measured by measuring the length of the crack in the Vickers indenter. Every value of the indentation fracture toughness is the mean of 10 measured toughness of the fracture. Indentation fracture toughness was calculated by the following equation [39]:

(2) $$K_{c}=0.018{\left(E/H_{v}\right)}^{0.5}\left(P/c^{1.5}\right)$$

## 3. Results and Discussion

**Figure 1:**
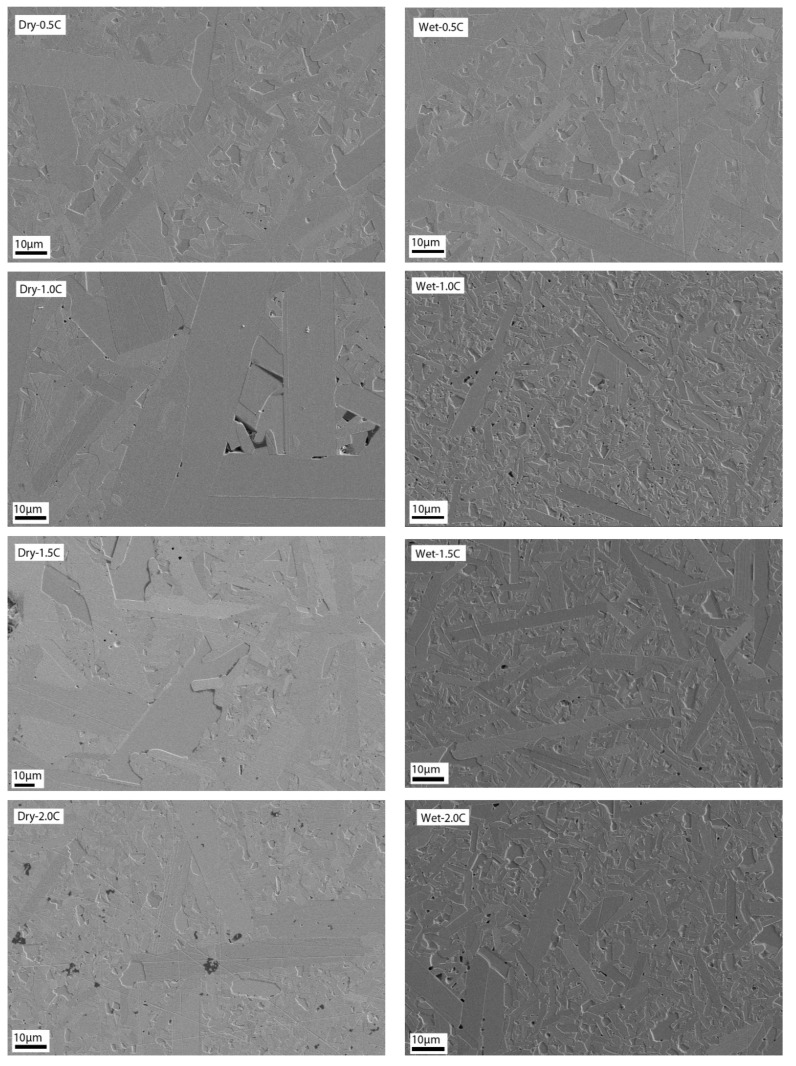

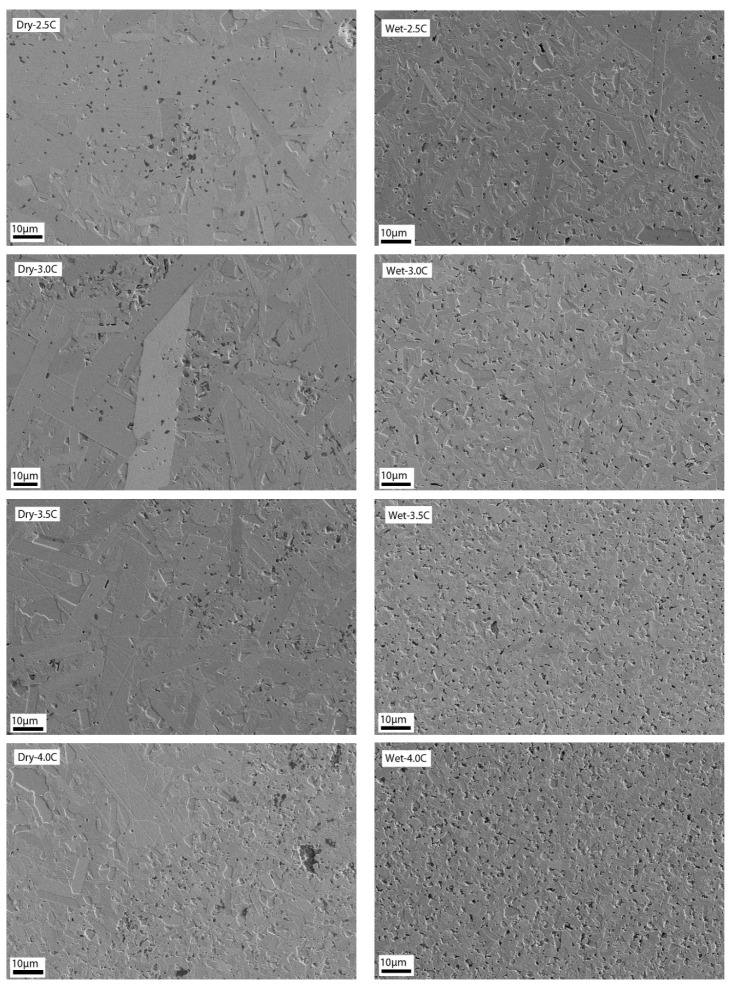

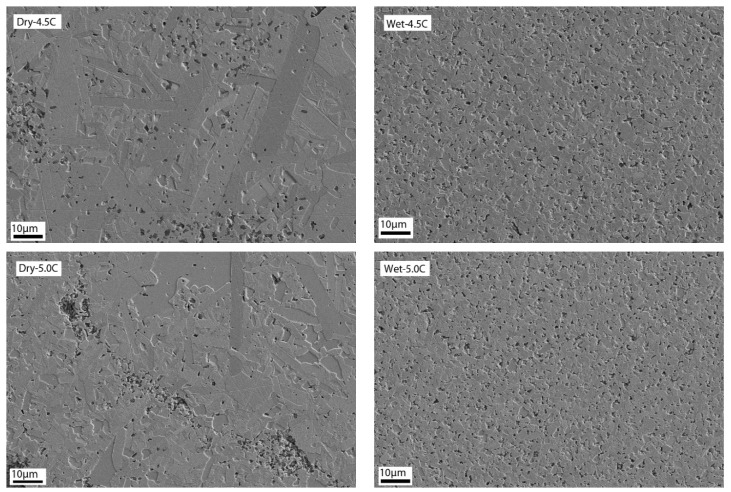
Field emission scanning electron microscope (FESEM) images for dry mixed–wet mixed SiC ceramics.

Figure 1 shows the FESEM images of the dry mixed (left side) and wet mixed (right side) series of SiC samples, and the average grain sizes of samples can be seen in Table 2. For 0.5 wt.% C, both dry and wet mixed samples showed similar microstructures with different grain sizes; the dry mixed sample had 4.4 μm average grains, whereas wet mixed sample had average grain size of 3.54 μm. With increasing the carbon content, dry mixed and wet mixed samples showed different morphology. Other than 0.5 wt.% C samples, dry mixed samples showed poor mixing, and wet mixing samples were well dispersed.

For 1.0 wt.% (8.79 μm) and 1.5 wt.% C (10.74 μm) dry mixed samples, large grain sizes and irregular shape porosities were observed. With increasing the carbon content from 2.0 to 5.0 wt.%, surplus carbon started appearing on the FESEM images. All samples had large, elongated grains and large quantity of porosity. Additionally, the images showed that the dry mixing method was not the way to have well disperse, similar grains.

On the other hand, wet mixed sample with 1.0 wt.% C (3.79 μm) had some slight porosity. The pores tented to decrease until the specimen reached the full density. Wet mixed 1.5 wt.% C (4.11 μm) sample have reached almost full density with only occasional small pores; it can be suggested that addition of carbon was enough to remove oxide layer. Both 1.0 and 1.5 wt.% samples had combined with small equiaxed grains and some elongation of grains. As seen in Table 2, average grain size increased until 1.5 wt.% C addition since there were less pores to prevent grain growth. Here, 2.0 and 2.5 wt.% C samples showed similar grain shapes with 1.0 and 1.5 wt.% samples but they had some porosity. By increasing the carbon content from 3.0 to 5.0 wt.%, grain sizes decreased (2.80–1.75 μm), and they showed more equiaxed grain shapes; it can be suggested that surplus carbon inhibited the elongated grain growth of silicon carbide and showed significant amounts of porosity.

**Figure 2:**
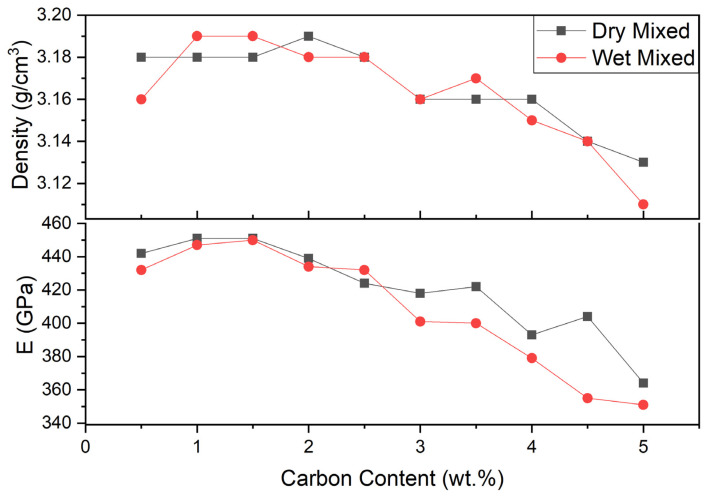
Dry–wet mixed SiC samples density and elastic modulus vs. carbon content.

The densities of the dry mixing and wet mixing series samples were determined using Archimedes’ method. They are shown below in Table 2. In addition, effects of carbon content on dry mixed and wet mixed SiC density and elastic modulus can be seen in Figure 2. When compared with the theoretical density of SiC, relative density values of dry and wet mixed samples series are between 96% and >99%. The oxygen impurities on the SiC powder inhibit to achieve fully dense SiC ceramics. In order to produce highly dense SiC ceramics, the same amount of B_4_C (0.5 wt.%) and 0.5–5.0 wt.% C were added to SiC. Addition of 0.5–2.5 wt.% carbon showed higher than 99% relative density. However, addition of carbon after 2.5 wt.% decreased the relative densities of samples.

Looking at the wet mixed series sample densities, it was clear that addition of 0.5 wt.% carbon was insufficient for removing the surface oxygen and reached the full densification. Addition of 1.0–1.5 wt.% carbon was enough carbon to efficiently removed the oxide layer from SiC surface, so that the density increased but as carbonaceous inclusions do not leave anything in the microstructure.

For all series, with higher levels of carbon, there is more than enough carbon to react with and eliminate oxygen and surplus carbon remained. Since the theoretical density of C is less than SiC and excess carbon caused porosities in the structure, the density of the samples decreased. Addition of less or large amount of carbon affects the SiC properties and prevents it from having high density. Maitre et. al. sintered SiC without sintering aids at 1950 °C under applied pressure of 100 MPa for 5 min by SPS. Despite the very high pressure at the same sintering temperature, only 97.5% density was achieved [18].

Elastic modulus is especially an important property for ceramics and can be used in special applications like SiC, since it tells when the material will deform. The material may require greater force to deform when the material has higher elastic modulus. Elastic modulus of dry mixed and wet mixed series can be seen in Table 2. For the dry mixing series, samples elastic modulus first increased with increasing the carbon content from 0.5 to 1.5 wt.% C. Continuing to add carbon, the elastic modulus of ceramic samples decreased. The elastic modulus values changed between 442 and 363 GPa for different carbon addition. The highest elastic modulus value achieved was 451 GPa at 1.0 and 1.5 wt.% C samples. The lowest elastic modulus value obtained was 363 GPa at 5.0 wt.% C.

The elastic modulus of wet mixed series changed between 432 and 351 GPa. The elastic modulus had the same trend with the dry mixed samples. The values first increased with increasing the carbon content, then decreased with increasing carbon addition. The highest elastic modulus value was obtained at 1.5 wt.% C, which was 450 GPa. The lowest elastic modulus value was obtained again at 5.0 wt% C, which was 351 GPa.

It was clear that there was a relationship between the density and the elastic module. Results showed that the elastic modulus increased with increasing the density of the samples. This result was found because of two reasons. First, carbon has lower elastic modulus value, so the surplus carbon affect the SiC ceramics, and it reduced the elastic modulus of dense SiC. Second, addition of carbon from 0.5 to 1.5 wt.% C helped to reduce porosity and SiC reached the high elastic modulus, however, addition of more than 1.5 wt.% of carbon caused porosity. Due to the porous structure, elastic modulus decreased. To achieve high dense SiC with high elastic modulus, sufficiently carbon should be added.

**Figure 3:**
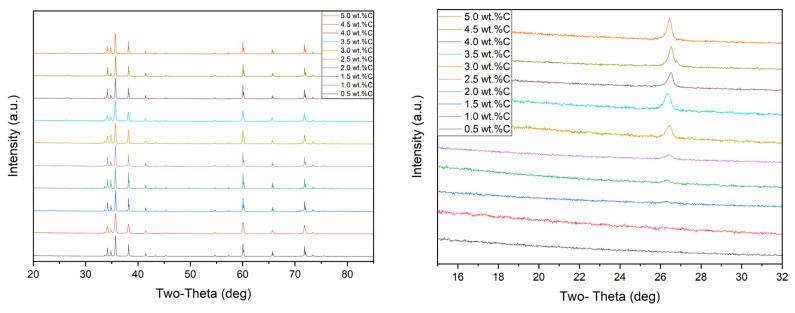
The phase identification of the XRD patterns of dry mixed SiC with different amounts of carbon addition (**left**), an enlarged view (2θ = 15°–32°) of X-ray diffraction patterns of dry mixed SiC (**right**).

**Figure 4:**
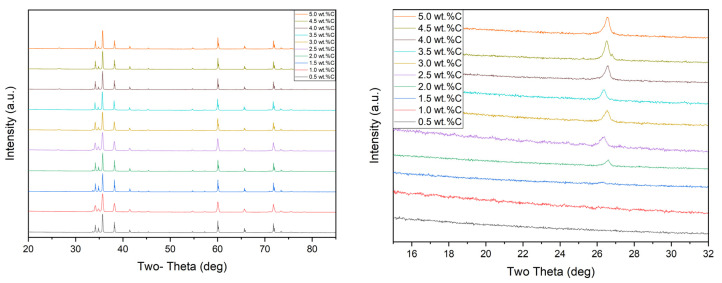
The phase identification of the XRD patterns of wet mixed SiC with different amounts of carbon addition (**left**), an enlarged view (2θ = 15° to 32°) of X-ray diffraction patterns of wet mixed SiC (**right**).

Figure 3 shows the phase identification of the XRD patterns of dry mixed dense SiC samples with different amounts of carbon addition and expanded view of XRD patterns 2θ = 15° to 32°, and Figure 4 shows the phase identification of the XRD patterns of wet mixed dense SiC samples with different amounts of carbon addition and expanded view of XRD patterns 2θ = 15° to 32°. The XRD pattern for the dense SiC specimens was matched with SiC patterns, and some samples had a low intensity broad peak that matched with carbon. Silicon carbide’s highest intensity peaks were located at ~36.0° 2θ and ~60.0° 2θ. Excluding SiC and C, the XRD pattern did not show any other types of contamination. It means that impurities were not introduced to the powder during the mixing process. For all series, samples showed similar patterns; only difference was that 0.5–1.5 wt.% C series did not show free carbon peak, and starting from 2.0 to 5.0 wt.% C samples showed excess carbon peak when the additional carbon was increased; the residual carbon peak located at ~26.6° 2θ also increased.

Rietveld refinement (quantitative analysis) of the XRD patterns of the samples may be seen in Table 3. From the analysis, it can be seen that SiC had two polytypes 4H and 6H, some specimens had free carbon, and free B_4_C was not found in XRD analysis of any samples. For both series, from 0.5 to 1.5 wt.% C samples did not have any excess carbon; it showed that all addition carbon was reached with oxygen. Free carbon started to appear when more carbon was added. The same amounts of carbon were added to dry mixed and wet mixed samples, but when looking at the free carbon’s amount, it can be seen that dry mixed samples had more surplus carbon in dense SiC. It may suggest that poor mixing (dry mixing) caused these results. It was clearly seen in FESEM images that specimens had agglomerates carbon.

Looking at the phase fraction of 4H/6H, we see that when samples had mainly 4H polytype, sample had larger averages grain sizes. It can be seen in Table 3. Dry mixed samples had mostly 4H polytype. Especially, 1.5 wt.% C samples had 71.9% 4H-SiC, and it had average grain sizes of 10.74 µm.

For the wet mixed samples, with increasing the carbon content from 0.5 to 1.5 wt.%, the amount of 4H polytype was higher than 6H polytype. In addition, the average grain size was enhanced until 1.5 wt.% C. However, as the amount of added carbon increased, both the average grain size and the 4H ratio decreased. Ayguzer Yasar et al. also was mentioned that 4H polytype displayed elongated grains, whereas 6H polytype showed small equiaxed grains [8]. Liu et al. sintered SiC with different amounts of carbon and boric acid without pressure at 2150 °C for half an hour. Despite the high sintering temperature, the density of the samples was less than 99%. Moreover, even in the sample with the highest density with lowest free carbon, there was still free carbon. The free carbon problem could not be eliminated, which affects the properties of SiC [40].

**Figure 5:**
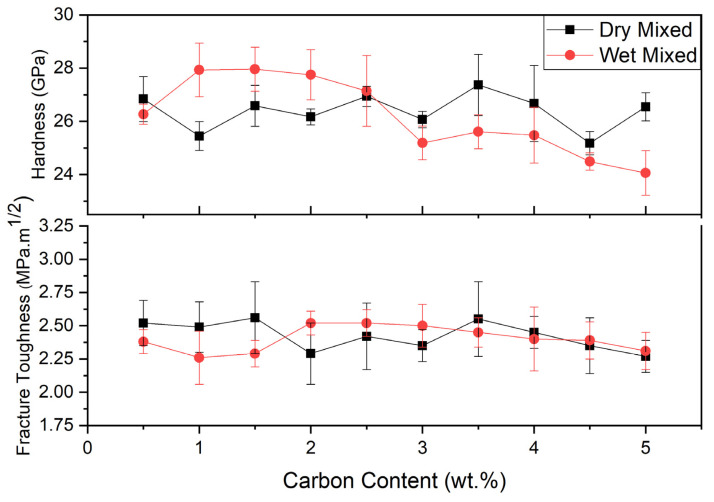
Dry mixed and wet mixed SiC samples hardness and fracture toughness.

Hardness and calculated fracture toughness values can be seen in Table 4 and Figure 5, respectively, for dry mixed and wet mixed dense SiC. Dry mixed SiC samples hardness values changed from 25.18 to 27.37 GPa. In addition, calculated fracture toughness values ranged from 2.27 to 2.56 MPa.m^1/2^. However, due to the poor mixing, carbon was not well distributed, so it was hard to make inferences about effect of carbon content.

Wet mixed SiC samples hardness values changed from 24.06 to 27.96 GPa. Having oxide layer on the SiC had negative effect on hardness of SiC ceramics. Since 0.5 wt.% C was not enough to remove all oxygen, SiC did not reach high hardness. However, 1.5 wt.% C addition was enough to remove oxide layer, hardness reached the highest value. Hardness increased from 26.27 to 27.96 GPa when the carbon content increased from 0.5 to 1.5 wt.%. Then with increasing the carbon content from 2.0 to 5.0 wt.%, hardness reduced from 27.75 to 25.48 GPa. Since carbon’s hardness is lower than SiC, surplus carbon caused drop in the hardness value of SiC ceramics. Excess carbon acts like oxygen, and it has detrimental effect on SiC. Sufficient carbon should be added to SiC in order to improve the properties of SiC. To compare the hardness values, in the study of Lomello et al., SiC samples were sintered at 1900 °C with a high applied pressure of 70 MPa. However, a hardness value of maximum 25 GPa could be obtained. This was because the sintering temperature was not sufficient, and the samples only reached 96% density, resulting in a lower hardness value [19].

Fracture toughness values vary in between 2.26 and 2.52 MPa.m^1/2^. Silicon carbide has a low fracture toughness, and the addition of different amounts of carbon has not been sufficiently modified to improve this feature.

## 4. Conclusions

In this study, to produce SiC ceramics, SiC powder mixed addition of 0.5 wt.% B_4_C and 0.5–5.0 wt.% C with dry mixing (specktromill) and wet mixing (ball milling) was done and samples were sintered at 1950 °C under applied pressure of 50 MPa for 5 min by SPS. Mixing method was found to be significant in properties of SiC ceramics. Dry mixing method showed poor mixing and detrimental effect of microstructural and mechanical properties of SiC ceramics. Samples mixed with dry mixing showed large grain sizes and large irregular shape pores. On the other hand, wet mixing showed well distribution of carbon. The highest density (99%) was reached in the sample with 1.5% C addition with wet milling method. The highest hardness and elastic modulus were achieved at 1.5 wt.% C, and the values were 27.96 and 450 GPa, respectively. Rietveld analysis showed that the samples had mainly 6H polytype when the high density was reached. In addition, it was found from the results of the analysis that the 4H polytype caused large elongated grains, whereas the 6H polytype caused small coaxial grains. Last but not least, 1.5 wt.% C addition was sufficient to remove the oxide layer from SiC and improve the properties of dense SiC ceramics. It has been observed that excess carbon affects the material as bad as oxygen. Surplus carbon inhibited to achieve high density with high elastic modulus and hardness.

## Figures and Tables

**Table 1:** SiC powder characteristics.

α-SiC Acheson Type Mainly 6H Polytype		
Total carbon		28.5–29.5 wt.%
Impurity	O	1.7 wt.%
	Fe	0.04 wt.%
	Al	0.03 wt.%
	Ca	0.003 wt.%
Specific surface area		23–26 m^2^/g
Particle size distribution	D 90%	0.76 µm
	D 50%	0.42 µm
	D 10%	0.22 µm

**Table 2:** The density, average grain size, and elastic modulus of dry mixed–wet mixed dense SiC.

	Density (g/cm^3^)		Average Grain Size (std. dev.) (µm)		E (GPa)	
Samples	Dry Mixing	Wet Mixing	Dry Mixing	Wet Mixing	Dry Mixing	Wet Mixing
0.5C	3.18	3.16	4.44 (0.88)	3.54 (0.96)	442	432
1.0C	3.18	3.19	8.79 (3.17)	3.79 (0.70)	451	447
1.5C	3.18	3.19	10.74 (3.03)	4.11 (0.61)	451	450
2.0C	3.19	3.18	2.84 (0.51)	4.05 (0.90)	439	434
2.5C	3.18	3.18	5.74 (1.28)	3.99 (1.40)	424	432
3.0C	3.16	3.16	5.82 (1.19)	2.80 (0.95)	418	401
3.5C	3.16	3.17	5.55 (2.13)	2.22 (0.41)	422	400
4.0C	3.16	3.15	3.23 (0.78)	2.17 (0.40)	393	379
4.5C	3.14	3.14	6.14 (1.78)	2.06 (0.57)	404	355
5.0C	3.13	3.11	4.95 (1.19)	1.75 (0.36)	364	351

**Table 3:** Rietveld refinement (quantitative analysis) of dry mixed–wet mixed SiC samples.

	Dry Mixing				Wet Mixing			
Samples	Phase Fraction 4H (%)	Phase Fraction 6H (%)	Phase Fraction C-2H (%)	Average Grain Size (std. dev.) (µm)	Phase Fraction 4H (%)	Phase Fraction 6H (%)	Phase Fraction C-2H (%)	Average Grain Size (std. dev.) (µm)
0.5C	50.3 (0.2)	49.7 (0.2)	–	4.44 (0.88)	45.0 (0.4)	55.0 (0.4)	–	3.54 (0.96)
1.0C	69.7 (0.5)	30.3 (0.3)	–	8.79 (3.17)	48.1 (0.2)	51.9 (0.2)	–	3.79 (0.70)
1.5C	71.9 (0.2)	28.1 (0.3)	–	10.74 (3.03)	57.6 (0.6)	42.4 (0.5)	–	4.11 (0.61)
2.0C	37.2 (0.2)	61.9 (0.2)	0.9 (0.2)	2.84 (0.51)	50.1 (0.2)	49.5 (0.3)	0.4 (0.2)	4.05 (0.90)
2.5C	53.5 (0.2)	45.2 (0.3)	1.3 (0.2)	5.74 (1.28)	49.2 (0.3)	50.0 (0.4)	0.8 (0.2)	3.99 (1.40)
3.0C	54.1 (0.3)	43.8 (0.3)	2.1 (0.2)	5.82 (1.19)	17.2 (0.2)	81.2 (0.4)	1.6 (0.2)	2.80 (0.95)
3.5C	52.9 (0.2)	44.7 (0.2)	2.4 (0.2)	5.55 (2.13)	17.6 (0.2)	80.3 (0.3)	2.1 (0.1)	2.22 (0.41)
4.0C	47.4 (0.2)	49.6 (0.4)	3.0 (0.3)	3.23 (0.78)	13.5 (0.2)	83.8 (0.3)	2.7 (0.2)	2.17 (0.40)
4.5C	55.2 (0.2)	41.4 (0.2)	3.4 (0.1)	6.14 (1.78)	13.2 (0.2)	83.7 (0.4)	3.1 (0.1)	2.06 (0.57)
5.0C	51.3 (0.2)	44.6 (0.3)	4.1 (0.2)	4.95 (1.19)	10.3 (0.2)	85.9 (0.3)	3.8 (0.2)	1.75 (0.36)

**Table 4:** Dry mixed and wet mixed SiC samples hardness and fracture toughness values.

	Hardness (GPa) (std. dev.)		Fracture Toughness (MPa.m^1/2^) (std. dev.)	
Samples	Dry Mixing	Wet Mixing	Dry Mixing	Wet Mixing
0.5C	26.84 (0.85)	26.27 (0.38)	2.52 (0.17)	2.38 (0.09)
1.0C	25.45 (0.54)	27.93 (1.01)	2.49 (0.19)	2.26 (0.20)
1.5C	26.58 (0.77)	27.96 (0.83)	2.56 (0.27)	2.29 (0.10)
2.0C	26.17 (0.30)	27.75 (0.94)	2.29 (0.23)	2.52 (0.09)
2.5C	26.94 (0.38)	27.14 (1.33)	2.42 (0.25)	2.52 (0.10)
3.0C	26.07 (0.31)	25.19 (0.63)	2.35 (0.12)	2.50 (0.16)
3.5C	27.37 (1.15)	25.61 (0.64)	2.55 (0.28)	2.45 (0.11)
4.0C	26.67 (1.43)	25.48 (1.05)	2.45 (0.12)	2.40 (0.24)
4.5C	25.18 (0.44)	24.49 (0.33)	2.35 (0.21)	2.39 (0.14)
5.0C	26.55 (0.53)	24.06 (0.84)	2.27 (0.12)	2.31 (0.14)
